# Colorectal self-expanding metal stent insertion using a small-caliber tapered transparent hood and electrolyte-free gel

**DOI:** 10.1055/a-2058-8760

**Published:** 2023-04-12

**Authors:** Tatsuma Nomura, Shinya Sugimoto, Taishi Temma, Jun Oyamada, Keiichi Ito, Akira Kamei

**Affiliations:** 1 Department of Gastroenterology, Ise Red Cross Hospital, Ise, Mie, Japan; 2 Department of Gastroenterology, Mie Prefectural Shima Hospital, Shima, Mie, Japan

Colorectal self-expanding metal stent insertion is a useful procedure to treat acute colorectal obstruction caused by advanced colorectal cancer; however, guidewire perforation has been reported to occur as a result of inadequate endoscopic visibility 1 2 . It is therefore important to maintain endoscopic visibility of the stricture to allow safe insertion of the colorectal stent. A method to maintain endoscopic visibility using an electrolyte-free gel (Viscoclear; Otsuka Pharmaceutical Factory) has recently been reported 3 4 ; however, it is difficult to maintain proper endoscopic visibility when the endoscopic hood has a large volume, as in the case of a conventional transparent hood. We previously reported a technique to obtain an appropriate field of view under saline immersion conditions using a calibrated, small-caliber tip, transparent hood (CAST hood; TOP, Tokyo, Japan) that has a tapered tip ( Fig. 1 ) 5 . Herein, we report the use of a novel technique for colorectal stent insertion using this small-caliber tapered transparent hood and electrolyte-free gel ( Video 1 ).

**Fig. 1:**
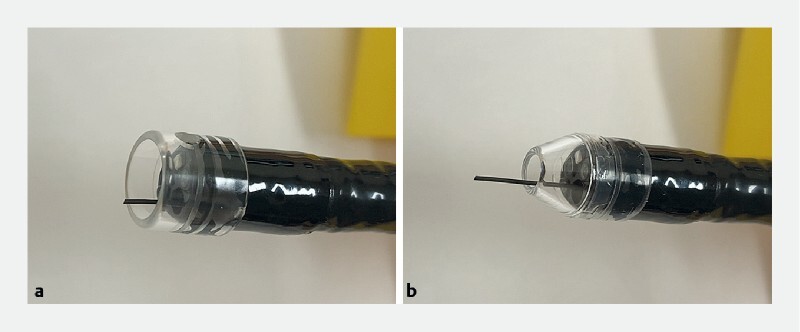
Photographs showing: **a** a cylindrical transparent hood and straight guidewire; **b** the small-caliber tapered hood and straight guidewire.

**Video 1** Stent insertion to treat a colonic stricture using a small-caliber tapered transparent hood.


The patient was an 84-year-old woman with acute colorectal obstruction due to advanced colorectal cancer of the sigmoid colon. We inserted a colonic stent to decompress the obstruction. To maintain endoscopic visibility, a transparent hood was placed on the endoscope tip, and endoscopy was performed using an electrolyte-free gel; however, due to the severity of the stricture, endoscopic visualization with the standard hood was restricted ( Fig. 2 a ). The tapered tip of the new transparent hood limited the amount of blood that could enter the tip attachment, and appropriate endoscopic visibility was maintained using this hood with the gel ( Fig. 2 b ). Central stricture of the tumor was confirmed. A guidewire was placed through the colonic stricture from the oral side and a colonic stent was successfully inserted ( Fig. 2 c, d ).

**Fig. 2:**
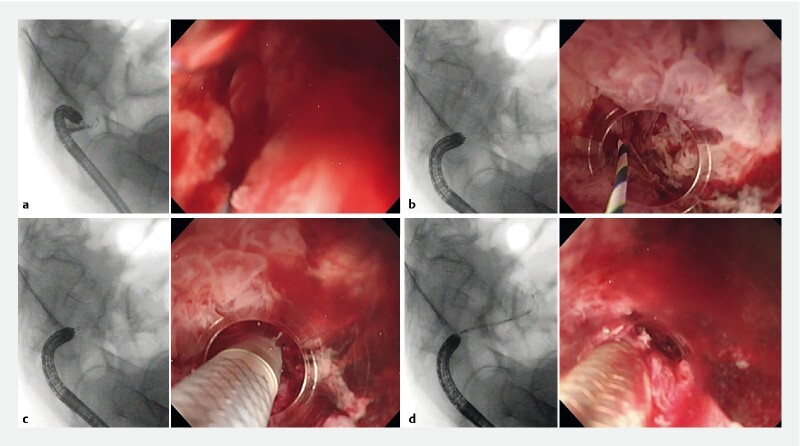
Paired fluoroscopic and endoscopic images showing: **a** the presence of a colonic tumor stricture that made insertion of a guidewire difficult using the cylindrical transparent hood; **b** safe guidewire insertion when using the small-caliber tapered hood, which allowed the stricture to be more accurately visualized; **c** a colonic stent being inserted through the tapered hood; **d** the self-expanding metal stent in position, with good endoscopic visibility having been maintained.

Use of the small-caliber tapered transparent hood and gel is a reliable method to maintain adequate endoscopic vision and ensure safe colonic stent insertion.

Endoscopy_UCTN_Code_TTT_1AQ_2AF

## Notes

Endoscopy E-Videos

https://eref.thieme.de/e-videos
*E-Videos* is an online section of the journal *Endoscopy* , reporting on interesting cases and new techniques in gastroenterological endoscopy. All papers include a high-quality video and are published with a Creative Commons CC-BY license. Endoscopy E-Videos qualify for HINARI discounts and waivers and eligibility is automatically checked during the submission process. We grant 100% waivers to articles whose corresponding authors are based in Group A countries and 50% waivers to those who are based in Group B countries as classified by Research4Life (see: https://www.research4life.org/access/eligibility/ ). This section has its own submission website at https://mc.manuscriptcentral.com/e-videos
